# Optic Neuritis Caused by Rathke's Cleft Cyst in Young Adult

**DOI:** 10.1155/2014/204762

**Published:** 2014-06-19

**Authors:** Namie Kobayashi, Toshiyuki Oshitari, Kentaro Kobayashi, Takatsugu Onoda, Hidetoshi Ikeda, Emiko Adachi-Usami

**Affiliations:** ^1^Southern Tohoku Research Institute for Neuroscience, Southern Tohoku General Hospital, 7-155 Yatsuyamada, Fukushima, Koriyama City 963-8563, Japan; ^2^Southern Tohoku Ophthalmological Clinic, 7-166 Yatsuyamada, Fukushima, Koriyama City 963-8052, Japan; ^3^Department of Ophthalmology and Visual Science, Chiba University Graduate School of Medicine, Inohana 1-8-1, Chiba, Chuo-ku 260-8670, Japan; ^4^Southern Tohoku Research Institute for Neuroscience, Research Institute for Pituitary Disease, Southern Tohoku General Hospital, 7-155 Yatsuyamada, Fukushima, Koriyama City 963-8563, Japan

## Abstract

We report a case of right optic neuritis caused by Rathke's cleft cyst (RCC) in a young adult. A 15-year-old boy presented with reduced visual acuity in the right eye. He was diagnosed with optic neuritis in the right eye 4 years earlier at other clinics before he was referred to our department. During our one-year examinations, the cause of the reduced vision in his right eye could not be determined conclusively. At the age of 17 years, a RCC was detected by a neurosurgeon who specialized in hypophyseal diseases. He underwent microscopic transsphenoidal resection of the cyst, and his vision recovered to 1.2 and he has had no recurrence for at least 9 months. We suggest that repeated rupturing of the RCC was the cause of the optic neuritis, and a RCC can be successfully treated by surgery even after 3 years of optic neuritis.

## 1. Introduction

Rathke's cleft cysts (RCCs) are nonneoplastic, sellar, or suprasellar epithelial cysts of the pituitary. RCCs usually present in the 4th and 5th decade of life and are typically located in the sella, with suprasellar extension in more than half of the cases. The vast majority of RCCs are asymptomatic and a spontaneous remission may occasionally occur, except for the large cysts that can exert pressure on the surrounding structures comprising the pituitary gland and chiasm [1–3]. RCCs are not easily found or demonstrated by the different types of imaging techniques and are often overlooked. We describe a 15-year-old boy who had been followed up as a case of idiopathic optic neuritis by other institutions for 4 years.

## 2. Case Report

**Figure 1:**
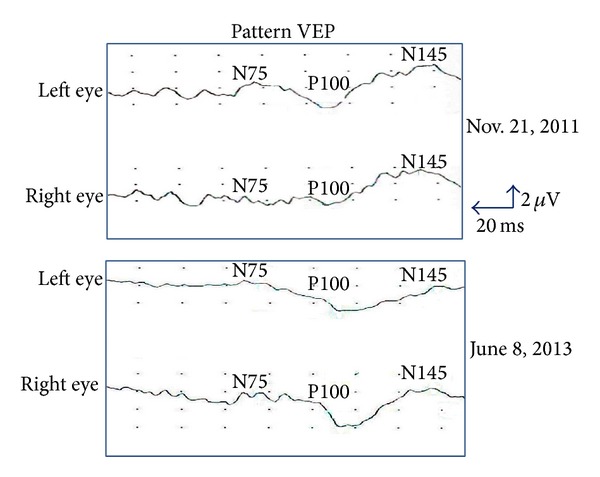
Transient pattern VEP before (upper two records) and after (lower two records) resection of a RCC. The P100 component was nonrecordable with stimulation of the right affected eye. The amplitude and peak latency recovered to the same level of the one with left nonaffected eye stimulation after surgery.

A 15-year-old boy who was treated for optic neuritis for 4 years was referred to our clinic on November 17, 2011, because the vision in his right eye was still poor in spite of steroid therapy. At the initial visit, the visual acuity of his right eye was counting fingers, and the critical flicker frequency and visual field were nonmeasurable. The relative afferent pupillary defect test was positive, and the pattern visually evoked potential (VEP; Figure 1, upper half) was nonrecordable. The optic disc appeared slightly pale, and the fluorescein angiography showed hyperfluorescence of the disc. The left eye was completely normal. Tests for the aquaporin-4 antibody and mitochondria DNA 11778 were negative. The blood cell counts and serum biochemical findings were within normal limits. Computed tomography (CT) and magnetic resonance imaging (MRI) of the brain were also negative.

**Figure 2:**
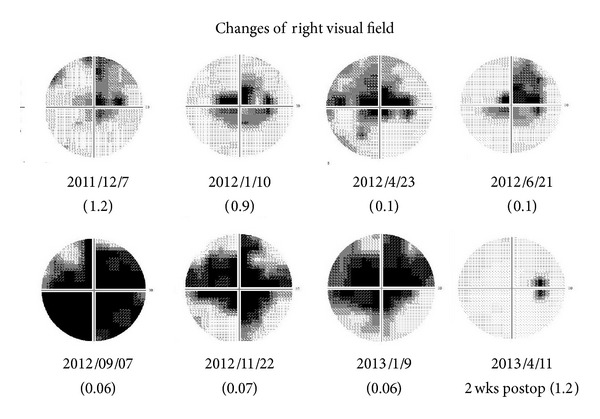
Time course of HVF 30-2 in the right affected eye. Before surgery, a central scotoma is present, and it gradually enlarged with increasing time. It is not present after the surgery.

With a tentative diagnosis of right optic neuritis, we started steroid pulse therapy, but the visual acuity did not improve significantly. Humphrey visual field (HVF) 30-2 tests showed a central scotoma whose density increased with increasing time (Figure 2). Subsequently, he complained of headaches and urinary incontinence. However, urological tests showed no abnormalities. He was regularly followed up in the neurological clinic.

**Figure 3:**
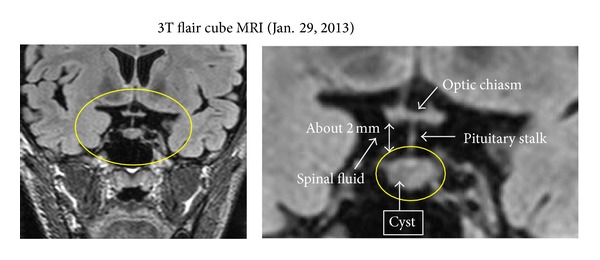
The 3T flair cube MRI taken on January 29, 2013. The MRI images demonstrated a RCC in the right pituitary region.

In February 2013, he reported that he had severe headache with nausea, and he was referred to our Neurosurgery Department for a detailed investigation of the pituitary gland. The neuroimaging examinations showed a RCC (Figure 3), and a microscopic transsphenoidal resection of the cyst was performed on March 26, 2013. Most of the specimens obtained by surgery composed of pituitary tissue with inflammatory cell infiltration. In a part of these specimens, epithelial cell linings which include goblet cells and microvilli were observed. These pathological findings were consistent with RCC.

On April 11, 2013, the right visual acuity had improved from 0.03 to 1.2, and no scotoma was found in the HVF. In addition, the VEPs returned to normal size and configuration (Figure 1, lower half).

His vision has remained at 1.2 up to this final examination on January 12, 2014.

## 3. Discussion

A RCC is sometimes difficult to detect by CT or MRI unless the cyst is large. In addition, a thinning of the bone around the sella tunica may not be apparent in young patients. Generally, the diagnosis is missed unless there is an alteration of endocrine function such as hyperprolactinemia. It is known that a RCC cyst on the pituitary stalk can cause visual disturbances, mainly bitemporal hemianopia [4]. Optic neuritis can also accompany a RCC [5].

The prognosis depends on the duration of symptoms and whether there is evidence of optic nerve damage at the time of presentation [6]. Patients with a shorter duration of symptoms and signs and with normal optic nerves have better visual prognosis. The severity of the preoperative visual disturbances, however, does not always predict the postoperative visual outcomes, and even patients with severe preoperative visual disturbances often have remarkable postoperative improvements.

Our case was a 15-year-old boy who was referred to our clinic after examinations by several ophthalmologists. He had a history of blurred vision which he had been told was optic neuritis or cat scratch eye disease since he was 11 years old. During the preceding 4 years, he had been treated with steroid pulse therapy three times but there was no improvement of his vision. Because the brain CT and MRI performed at the Neurological Department did not demonstrate a RCC at the initial examination, we also tried steroid pulse therapy which was ineffective.

Finally, a RCC was found by a specialist of pituitary diseases. As shown, the images of the RCC were very indistinct and difficult to detect. In the end, surgery was performed 6 years after the onset of the symptoms of optic neuritis. His visual acuity and central scotoma improved to normal almost immediately after the surgery, and they have remained stable for at least 9 months.

Our case demonstrated that vision could recover to normal levels by surgery even after a long duration of severe vision decrease by optic nerve neuritis resulting from a RCC in a young patient. Bohnstedt et al. [4] have reported a similar case that had a rapid visual acuity improvement after surgery. They concluded that this could be secondary to ischemic optic neuropathy caused by localized compression. In our case, we suggest that the neuropathy was due to inflammation resulting from the leakage of the cyst material into the subarachnoid space after repeated RCC rupture.

In conclusion, we suggest that repeated rupturing of a RCC can cause optic neuritis and myelitis. The disease can be resolved by surgery even after a longer duration of severe vision disturbances. Thus, we recommend that RCC should be considered in any case of optic neuritis of unknown etiology.
